# Genetic regulation of major immunogenic protein accumulation in peanut seeds

**DOI:** 10.1007/s10142-026-01916-x

**Published:** 2026-06-11

**Authors:** Tariq Alam, Gautam Saripalli, Carolina Ballen-Taborda, Sachin Rustgi

**Affiliations:** 1 Department of Plant and Environmental Sciences, Clemson University Pee Dee Research and Education Center, Florence, SC 29506 USA; 2 School of Health Research, Clemson University, Clemson, SC 29634 USA; 3 Center for Human Genetics, Clemson University, Greenwood, SC 29646 USA

**Keywords:** Peanut, Seed-storage proteins, Allergy, Protein quantity loci, Immunogenic proteins

## Abstract

Peanut is a major oilseed crop in the U.S., which ranks third globally in production, with South Carolina ranking sixth nationally. Despite their economic and nutritional value, peanuts are unsuitable for 1–2% of the U.S. population due to allergic reaction, including anaphylaxis, making the development of reduced-allergen peanuts a priority. This study aimed to identify peanut lines with reduced Ara h1, h2, h3, and h6 levels and to elucidate their genetic regulation using genome-wide association studies (GWAS). Ninety-two accessions from the U.S. peanut mini-core collection were screened using ELISA and SDS-PAGE, and the resulting data were evaluated for association with 5,532 SNP markers. Substantial phenotypic diversity was observed. Twenty-three lines with extreme protein phenotypes were further evaluated by RP-UPLC, and nine by LC-MS. GWAS identified 165 marker-trait associations (MTAs) across raw and log-transformed datasets, with 13 MTAs common to both analyses. These MTAs were grouped into protein quantitative loci (PQLs), revealing six *trans*-PQLs and *cis*-PQLs for two of the five *Ara h* genes. Seed-expressed candidate transcription factors (TFs), including MYB, MYC, ERF, and bZIP, and pleiotropic PQLs were identified. Promoter analysis (1 kb upstream of transcription start site or TSS) of the corresponding *Ara h* genes revealed binding sites for TFs underlying these PQLs. We hypothesize that these TFs trans-regulate the *Ara h* genes either directly or through a regulatory cascade. These findings provide initial insights into the regulatory landscape of *Ara h* genes and will facilitate breeding for reduced allergen content in peanut.

**Supplementary Information:**

The online version contains supplementary material available at 10.1007/s10142-026-01916-x.

## Introduction

Peanut (*Arachis hypogaea* L.) is a legume used for human consumption and oil extraction. It is primarily grown in tropical and subtropical agroecological regions of the world, with an annual production of about 51.4 million metric tons in 2024 (USDA FAS^2025^). Globally, the amount of peanuts produced per hectare differs by country and region, with the United States yielding 46,352 hg/ha and China 38,706 hg/ha (FAO 2021). It is the fourth most crucial oilseed crop (after soybean, rapeseed, and sunflower) worldwide and serves as a vital source of protein for millions globally (Chenault et al. 2009). The high content of seed oil (~ 46–58%) and protein (~ 22–32%) make it a crucial crop in combating malnutrition and enhancing food security worldwide. Furthermore, peanut offers a wide array of health benefits, including a high content of cardioprotective oleic and linoleic acids, anti-aging resveratrol, dietary fiber, and folic acid, coupled with highly balanced proteins (Chen et al. 2016). It is consumed in many different forms like oil, butter, boiled peanuts, energy bars, candy and as a paste in food. Furthermore, the skin of peanuts is rich in antioxidants and dietary fiber (Ciftci and Suna 2022). While peanuts are nutritionally important, they are also one of the potent causes of allergies and therefore, have attracted the attention of researchers working in the different life sciences disciplines. While medical researchers are working to develop drugs to combat peanut allergy, plant breeders and geneticists are focused on developing reduced-allergen peanut varieties and understanding the genetic regulation of allergenic protein accumulation in peanut seeds.

The cultivated peanut is a segmental allotetraploid with two sub-genomes, ‘A’ and ‘B,’ with an aggregate size of ~ 2.7 Gb (Bertioli et al. 2016). The *Arachis* genus consists of 81 species, predominantly diploids, with chromosome count of 2*n* = 2x = 20 (Grabiele et al. 2012; Robledo et al. 2009), except for two tetraploid species, *A. hypogaea* and *A. monticola* (AABB-type genome; 2*n* = 4x = 40). Different sub-species in taxonomical classification include *hypogaea*, *hirsuta*, *fastigiata*, *vulgaris*, *aequatoriana*, and *peruviana* (Bertioli et al. 2011; Krapovickas et al. 2007, 2009). The available landraces and cultivars of peanut are categorized by variations in grain colors and dimensions, pod morphologies, and growth patterns (Krapovickas et al. 2007, 2009). *Arachis hypogaea* is known to evolved through a single recent hybridization-polyploidization-event between two diploid species (Bertioli et al. 2019). Genomic in situ hybridization indicates a possible immediate ancestral linkage between *A. monticola* and *A. hypogaea* (Seijo et al. 2007). Aside from differences in the agronomic attributes, cultivated peanuts and their wild counterparts also show a few cytogenetic and genetic modifications in their genomes following polyploidization (Fávero et al. 2006; Nielen et al. 2012; Samoluk et al. 2015).

Peanut allergy (PA) remains a widespread concern, associated with significant morbidity and mortality. It typically manifests around two years of age, with approximately 20% of affected children outgrowing the allergy over time (Hourihane 2011). The incidence of PA has increased in the United States over the past decade (Fig. S1). Peanut allergy is the leading cause of fatal anaphylactic reactions to foods in North America and the United Kingdom (Hourihane 2011). Notably, the incidence of anaphylaxis is three times higher in children with peanut and tree nut allergies compared to those with other food allergies (McWilliam et al. 2019; Sasaki et al. 2018).

To manage PA, individuals must exclude peanuts from their diet (Shah et al. 2019). The avoidance often extends to other tree nuts and legumes, limiting available protein sources, particularly for vegetarians and vegans. Avoiding peanuts is challenging due to their widespread use in processed foods, the risk of unintended contamination and the associated financial burden. Indeed, inadvertent peanut exposures have been reported in up to 12.4% of affected individuals annually (Cherkaoui et al. 2015). Currently, no therapies exist to prevent reactions to peanut exposure. In the event of accidental exposure, immediate administration of epinephrine is required, often alongside anti–mast cell agents such as antihistamines (Burks 2008; Capucilli et al. 2020; Cardona et al. 2020; Reber et al. 2017).Efforts to develop preventive therapies have led to the approval of oral immunotherapy, Palforzia™ (Vickery et al. 2019), and the monoclonal antibody XOLAIR^®^ (omalizumab; Wood et al. 2024). While effective, these therapies are expensive, highlighting the need for more accessible alternatives. One promising approach is the development of peanut genotypes with significantly reduced levels of immunogenic proteins, which could offer a cost-effective strategy for reducing allergic risk.

Out of 32 proteins known to accumulate in peanut seeds, 18, specifically Ara h1 to Ara h18 (with Ara h4 renamed Ara h3.02), are recognized as allergens (Pele 2010; Yu and Eghbali 2025). The proteins Ara h1, Ara h2, Ara h3, and Ara h6 are considered major allergens as they elicit immune reactions in ≥50% of peanut-sensitive individuals (Shah et al. 2019). Peanut seeds comprise 25% protein by weight, which includes 12–16% Ara h1, 5.9–9.3% Ara h2 and Ara h6, and 20% Ara h3. Genes encoding major allergens have been cloned in peanut, and the structure of gene families, as well as their spatial and temporal expression patterns, has been studied (Kang et al. 2007a, b; Konan et al. 2009; Chen et al. 2016). Although natural genetic diversity in allergenic protein content has been reported in peanut seeds (Kang et al. 2007a, b; Konan et al. 2009; Wu et al. 2016; Pandey et al. 2019; Rustgi et al. 2022; Meng et al. 2024; Alam and Rustgi 2025), these qualitative and quantitative differences in seed proteins have not been utilized as traits to map the genomic regions regulating protein-level variation, termed protein quantity loci (PQLs). Similar studies have been conducted in wheat, soybean, and pea, where PQLs were identified using quantitative and qualitative differences in protein accumulation in various plant tissues and used as traits to identify *cis*- and *trans*-regulatory genomic regions (Amiour et al. 2003; Santos et al. 2022; Liu et al. 2025; Bourgeois et al. 2011).

The U.S. peanut mini-core collection consists of 112 accessions representing ~ 1% of the entire U.S. peanut germplasm collection and capturing substantial diversity for agronomic and quality traits (Holbrook and Dong 2005). This panel serves as an important genetic resource for breeding programs aimed at improving resistance to insect pests and pathogens, tolerance to abiotic stresses, and end-use quality traits. It has also been widely used to investigate the genetic regulation of traits such as disease resistance (Chenault et al. 2010; Holbrook and Dong 2005; Sarkar et al. 2022), seed fatty acid content and composition (Dean et al. 2009; Wang et al. 2013), heat tolerance (Selvaraj et al. 2011; Ingole et al. 2025), and allergen content (Kang et al. 2007a, b; Rustgi et al. 2022; Meng et al. 2024).

Population structure, genetic diversity, and phylogenetic relationships have also been characterized for this panel (Barkley et al. 2007; Wang et al. 2011), making it suitable for linkage disequilibrium (LD)-based genome-wide association studies (GWAS). After removing genotypes exhibiting morphological heterogeneity, a subset of 104 accessions was formally registered as the U.S. Peanut Mini-Core Germplasm Collection (Chen et al. 2013).

The present study investigates PQLs controlling Ara h allergenic proteins in peanut. Genotypes from the U.S. peanut mini-core collection were utilized to identify genomic regions associated with variation in protein abundance using GWAS. Specifically, this study aimed to (1) screen 92 genetically unique accessions of the U.S. peanut mini-core collection to identify genotypes with reduced levels of the major immunogenic proteins Ara h1, Ara h2, Ara h3, and Ara h6, and (2) identify and characterize PQLs by using protein abundance as the phenotype in GWAS to uncover candidate genes underlying variation in allergenic protein content. To achieve this, widely used protein profiling approaches—including SDS-PAGE, ELISA, HPLC, and LC–MS—were employed to quantify allergenic proteins and identify low-immunogenic peanut genotypes within the mini-core collection. Both *cis*- and *trans*-acting QTL were identified. *Cis*-QTL were defined as MTAs in LD with the genes encoding Ara h proteins. Using this approach, *cis*-QTL for *Ara h1* (Chromosome 19; LD 1.52 Mb) and *Ara h3* (Chromosome 6; LD 1.79 Mb) were identified. In contrast, *trans*-QTL were defined as MTAs located outside these regions. Although multiple classes of genes were associated with the *trans*-QTL, particular emphasis was placed on transcription factors due to their well-established roles in trans-regulation of gene expression. Overall, this study provides valuable genetic resources in the form of peanut genotypes with reduced allergenic protein content, as well as genomic resources including SNP markers linked to allergen-associated loci. These findings will facilitate the development of peanut cultivars with reduced immunogenicity through molecular breeding. Furthermore, the identified candidate genes and *trans*-PQLs establish a foundation for future functional validation studies, including CRISPR-based genome editing and other molecular approaches to confirm their regulatory roles in controlling allergenic protein accumulation.

## Materials and methods

### Plant material

Seeds of the 92 non-redundant genotypes of the U.S. peanut mini-core collection (see Supplementary Table S1) were obtained from the USDA-ARS Plant Genetic Resources Conservation Unit in Griffin, GA (https://npgsweb.ars-grin.gov/gringlobal/search). Pure seeds of these lines were used for total seed protein extraction and sodium dodecyl sulfate polyacrylamide gel electrophoresis (SDS-PAGE). Major allergenic proteins (Ara h1, Ara h2, Ara h3, and Ara h6) were quantified using densitometric analysis and enzyme-linked immunosorbent assay (ELISA). Based on SDS-PAGE and ELISA results, a subset of 23 genotypes was selected for Reversed-Phase Ultra-Performance Liquid Chromatography (RP-UPLC). Data from these methods (SDS-PAGE, ELISA, and RP-UPLC) were then used to select nine lines for further analysis via liquid chromatography–mass spectrometry (LC-MS).

### Seed storage protein extraction

Total protein from peanut seeds was extracted following Moutete et al. (1995) with minor modifications. Briefly, the protein was extracted through following steps (i) peanut seed was split into two halves; the embryo half (stored in −80 °C for later use in propagation) and the cotyledon half (100–300 mg). The latter was flacked and ground into flour using TissueLyser II (Qiagen Inc., Germantown, MD, USA) to obtain peanut flour. (ii) The flour was defatted by supplementing it with 1 mL acetone and churning the mixture for 1 h at 4 °C using a rotary and pelleting it by centrifugation at 337 g (Eppendorf^®^ Centrifuge 5810R, 17.3 cm radius) for 30 s. (iii) Subsequently, the pellet obtained from the previous step was washed with diethyl ether until a clear washout solution appeared and then dried under vacuum for 5 min at 30 °C. (iv) The pellet was extracted with 0.1 M ammonium bicarbonate under constant shaking at 250 rpm for 2.5 h and centrifugation at 337 g (Eppendorf^®^ Centrifuge 5810R, 17.3 cm radius) for 10 min and the supernatant was collected and stored at 4 °C for later use.

### Protein quantitation assay

The concentration of total seed protein was determined using the Pierce™ BCA method according to the manufacturer’s instructions (Cat. #23225, Thermo Fisher Scientific Inc., Rockford, IL, USA). Briefly, 25 µL of each standard (protein of known concentration diluted to 20–2,000 µg/mL) and peanut sample was transferred to a microtiter plate. Subsequently, 200 µL of the Pierce reagent was added to each well, and the contents were mixed on a plate shaker for 30 s. The plate was then incubated at 37 °C for 30 min and allowed to return to room temperature before measuring absorbance at 562 nm using a Beckman Coulter DU 730 UV/Vis spectrophotometer. Protein concentrations in the samples were quantified by plotting their absorbance values against a standard curve generated using bovine serum albumin (BSA) standards of known concentration.

### Sodium dodecyl sulfate polyacrylamide gel electrophoresis (SDS-PAGE)

Peanut seed proteins were resolved using 12% denaturing SDS polyacrylamide gels following the procedure described by Fling and Gregerson (1986). Briefly, the gel consists of two layers, separating and stacking. A single separating gel comprise 4.2 mL acrylamide stock solution (30% acrylamide: 0.8% bis acrylamide; Cat #161 − 0154, Bio-Rad Laboratories, Hercules, CA, USA), 4.2 mL doubled distilled (dd) water, 3 mL 3 M Tris-HCl (pH 8.8), 120 µL 10% SDS, 120 µL 10% ammonium persulfate (APS), and 6 µL N, N,N′,N′-Tetramethylethylenediamine (TEMED). Whereas the stacking gel consists of 1 mL acrylamide solution (30% acrylamide: 0.8% bis acrylamide), 750 µL 1 M Tris-HCl (pH 6.8), 4.25 mL dd water, 60 µL of 10% SDS, 60 µL 10% APS, and 4 µL TEMED. The separating gel was casted first and the stacking gel was layered over it.

Electrophoresis was performed in a BioRad Mini-PROTEAN^®^ tetra-gel electrophoresis system (Cat# 1658004, Bio-Rad Laboratories, Hercules, CA, USA) utilizing a buffer comprised of 25 mM Tris-HCL, 192 mM Tricine, and 0.1% SDS at 120 volts for one hour and 20 min. Post-electrophoresis, the gels were stained in Colloidal Coomassie G-250 stain, following Neuhoff et al. (1988). Equal concentrations (5000 µg/mL) of proteins (determined using Pierce™ BCA protein assay) were loaded onto the gels. Before loading the samples were supplemented with 2X loading buffer [125 mM Tris-HCl (pH 6.8), 4% SDS, 20% glycerol, 0.02% bromophenol blue, 10% β-mercaptoethanol or 100 mM dithiothreitol DTT] and heat denatured at 100 °C for 5 min. A pre-stained protein molecular weight marker (Cat # 1610375, Bio-Rad Laboratories, Hercules, CA, USA) was loaded into each gel.

### Densitometric analysis

The densitometric quantification of protein bands was performed using ImageJ software, following Mejías et al. (2014). Briefly, the images were converted to 16-bit format and background subtraction was applied to determine the optical density (OD) of each protein band. The densitogram displayed the OD of each band as a peak, with the area under each peak correlating with the OD of the protein bands. A standard curve was prepared by loading known quantities of BSA onto gels and processing them in a similar manner, allowing for the translation of peak areas into protein quantities in micrograms (Mejías et al. 2014).

### Measurement of allergens by enzyme-linked immunosorbent assay (ELISA)

Major peanut allergens, Ara h1, Ara h2, Ara h3, and Ara h6, were quantified using commercial ELISA kits according to the manufacturer’s instructions (Indoor Biotechnologies Inc., Charlottesville, VA, USA). Briefly, the pre-coated 96-well plates were washed thrice with 1x wash buffer. After each wash, excess buffer was removed by inverting the plates upside down on paper towels. Subsequently, 100 µL of 1x assay buffer was added to each well, with an additional 80 µL added to wells A1 and B1. Standards for each protein (Ara h1, Ara h2, Ara h3, and Ara h6) were gently vortexed, and 20 µL were added to wells A1 and B1. Then, 100 µL from wells A1 and B1 were transferred to A2 and B2, mixed, and this process is repeated up to wells A10 and B10 to establish a dilution series. Wells A11, A12, B11, and B12 were left blank. Total seed-storage protein extracted from different peanut genotypes was diluted to an equal concentration and further diluted 1/40. Subsequently, 20 µL of each dilution was sampled in triplicate into the remaining wells. The plates were covered with aluminum seals and incubated for one hour and five minutes with constant shaking. The kits included allergen standards, monoclonal antibodies (MAbs; both primary and secondary biotinylated antibodies), and enzyme conjugates for the four major peanut allergens: Ara h1, Ara h2, Ara h3, and Ara h6. Optical density (OD) was measured at 450 nm using a spectrophotometer. Concentrations of all allergens were determined using standard curves. The detection limit for the ELISA tests ranged from 0.2 ng/mL for Ara h6 to 31.5 ng/mL for Ara h1.

### Reversed phase ultra-performance liquid chromatography (RP-UPLC)

Immunogenic proteins (Ara h1, Ara h2, Ara h3, and Ara h6) were also quantified using RP-UPLC following Singh et al. (2016) with minor modifications. A UPLC C18 column (Cortecs UPLC T3 1.6 μm 2.1 × 150 mm) was used and the column temperature was adjusted to room temperature for the analysis. The liquid chromatography was performed using a two-pump gradient system, with phase C, consisting of 0.04% TFA in water, and mobile phase D, comprised of 0.04% TFA in acetonitrile, to resolve samples. The flow rate was maintained at 0.13 mL/min and the injection volume to 2 µL. The detection was performed using a photodiode array (PDA) between 190 nm and 400 nm over a 25-minute run, and chromatograms were recorded at 280 nm. To confirm allergen peaks in the total seed storage protein samples, the samples were spiked with pure allergens prior to UPLC separation.

### Liquid chromatography-mass spectrometry (LC-MS/MS)

The samples were prepared by aliquoting 50 µL of the sample, which contained 50 µg of protein, into tubes. To this, 5 µL of 200 mg/mL Sodium Dodecyl Sulfate (SDS) and 5 µL of 200 mM Tris(2-carboxyethyl)phosphine (TCEP) were added. The mixture was then incubated at 55 °C for 15 min to facilitate the reduction of disulfide bonds. Following the reduction step, 5 µL of 400 mM Iodoacetamide (IAA) was added to the mixture. This was followed by an incubation at 23 °C for 30 min to allow for the alkylation of cysteine residues to prevent the reformation of disulfide bonds. Subsequently, the proteins were precipitated by adding 6.5 µL of 27.5% phosphoric acid and 500 µL of binding buffer (90% methanol, 100 mM triethylammonium bicarbonate (TEAB), 0.03% phosphoric acid) to the mixture. The mixture was then loaded onto S-Trap micro spin columns for further processing. This step is crucial for ensuring the proteins are properly precipitated and prepared for digestion. For protein digestion, 20 µL of 0.25 µg/µL trypsin protease was added to the sample. The sample was then incubated at 37 °C in a water bath for 13.5 h to allow for complete digestion of the proteins into peptides. This extended digestion time ensured thorough cleavage of the proteins. The peptides were then eluted from the S-Trap columns. Once eluted, the peptides were dried and reconstituted to a final protein equivalent concentration of 1 µg/µL. This reconstitution step prepared the peptides for analysis by UHPLC-MS/MS.

The prepared samples were analyzed by injecting 2 µg of the sample into an UHPLC-MS/MS system [Ultimate 3000 UHPLC system coupled to an Orbitrap Fusion Tribrid Mass Spectrometer with Electrospray Ionization (ESI) operated in positive ionization mode] for proteomic analysis. The column used for separation was a Waters™ ACQUITY UPLC^®^ Peptide CSH™ C18 column (130Å, 1.7 μm, 150 × 1 mm), which was maintained at 40 °C for analysis. The solvent gradient used for the UHPLC separation consisted of Solvent A (0.05% formic acid in water) and Solvent B (0.05% formic acid in acetonitrile). The mass spectrometer parameters for MS1 scans included a Monoisotopic Precursor Selection (MIPS) setting of “Peptide,” an intensity threshold of 1.5e^4^, and a charge state range of 2–7. Dynamic exclusion was set to exclude after one time within 15 s for a mass tolerance of 10 ppm, excluding isotopes.

Data analysis was performed using Proteome Discoverer 2.4.15. The workflow employed was designed for label-free quantification, utilizing the Total Protein Approach (TPA). The FASTA files used included ‘uniprotkb_arachis_hypogaea_2024_01_08_canon.fasta’ (containing 185,388 sequences and 65,593,124 residues) and ‘Ara.fasta’ (created from unique peptide sequences, containing 9 sequences and 3,782 residues). Protein quantification was based on the average intensity of the top three most abundant unique and razor peptides. Data filters applied included a requirement for at least two unique peptides per protein and exclusion of contaminants. Quality control (QC) checks were performed to ensure the accuracy and reliability of the method. Method QCs involved preparing and processing 1 mg/mL BSA, with results showing 80% protein coverage and 72 peptides, with a coefficient of variation (CV) of 2.48%. Instrument QCs involved analyzing a 2 pmol BSA digest once every ten samples, with results showing 85% protein coverage and 76 peptides, with a CV of 4.44%.

### Phylogenetic analysis, bar diagrams, descriptive statistics, and Pearson correlation coefficient

Phylogenetic analysis of the 92 accessions was conducted using protein traits estimated by densitometry and ELISA, as well as genotyping data, in JMP Pro 18. A hierarchial clustering algorithm using Ward’s method was applied to calculate distance matrices in JMP, and a univariate ANOVA was fitted using cluster assignments as groups. Concentrations of the four major allergenic proteins were visualized using bar charts created in MS Excel. Pearson correlations were calculated using the psych package in R. The Pearson correlation matrix, frequency distribution curves, and scatter plots were combined and presented in a single chart generated in R. Descriptive statistics, including the mean, standard deviation and coefficient of variation (CV%), were calculated using MS Excel for all the four Ara h protein traits estimated using both SDS-PAGE and ELISA. Only log-transformed values were used for descriptive statistics due to the presence of outliers in the raw data (see discussion).

### Genotyping data

Pre-curated genotyping data for the U.S. peanut mini-core collection used in this study were retrieved from PeanutBase (Otyama et al. 2019). A total of 15,987 SNPs generated using the 58 K Affymetrix SNP array were available. For the final GWAS analysis, 5,532 filtered SNPs were used after applying the following filters: minor allele frequency > 5%, heterozygosity < 10%, and missing data < 10%.

### Identification of marker trait associations (MTAs), PQLs, candidate genes (CGs), and expression pattern of CGs

For the identification of MTAs, two popular multi-locus models, BLINK and FarmCPU, were utilized. Both methods were implemented using GAPIT v 3.0 (Lipka et al. 2012). GWAS was conducted using both raw and log-transformed data for four proteins (Ara h1, Ara h2, Ara h3, and Ara h6) quantified through densitometric analysis of SDS–polyacrylamide gels and ELISA. Because the U.S. peanut core collection comprises highly diverse genotypes, protein content across genotypes exhibited a broad distribution. This variation was often influenced by single outlier genotypes that may carry unique modifier alleles regulating protein accumulation. To avoid excluding any genotypes from the analysis while minimizing the detection of false-positive MTAs, the protein data were log-transformed prior to analysis. Log transformation of the data was performed using Microsoft Excel. Important MTAs were identified using two different criteria: (i) MTAs that met Bonferroni threshold according to the formula α/total number of markers, where α = 0.05 and the total number of markers = 5,532. Therefore, the Bonferroni threshold was calculated as *P* = 0.05/5532 = 9.038 × 10^− 5^, or -log(9.038 × 10^− 5^) = 5.04, and (ii) MTAs with a suggestive significance threshold of −log10(P) ≥ 3 (*P* ≤ 0.001) (Bomireddy et al. 2024). LD for individual chromosomes was previously identified in an earlier study using the same U.S. peanut mini-core collection (Otyama et al. 2019). These LD regions were used to identify the potential QTL(s) associated with specific proteins, which were designated as PQLs. Furthermore, the genomic locations of the known *Ara h1*, *Ara h2*, *Ara h3*, and *Ara h6* genes were extracted and plotted on the chromosome maps to examine their proximity to the identified MTAs. If any of the known *Ara h* genes were located within the same LD region as the MTAs, such MTAs were considered *cis*-PQLs (PQLs associated with the structural genes for the major peanut allergens—Ara h1, Ara h2, Ara h3, and Ara h6). In contrast, MTAs located outside of the LD blocks surrounding the *Ara h1*, *Ara h2*, *Ara h3*, and *Ara h6* genes were considered *trans*-PQLs (Morgan et al. 2020).

For identifying the PQLs based on LD, only MTAs (at least one out of two or more) that were commonly identified using both raw data and log-transformed data were considered, to minimize the possibility of false-positive QTLs. Furthermore, only *trans*-PQLs (not co-locating with known *Ara h* genes) were used for identifying candidate genes (CGs). For candidate gene identification, the physical coordinates of the MTAs within the LD region were used as input in the BioMart tool of Ensembl Plants, and the accession IDs of CGs were extracted along with the protein domains encoded by these genes. Only CGs encoding transcription factors or protein domains matching those of the known *Ara h* genes were further used for in silico expression analysis using the online database ArachisMine (https://mines.legumeinfo.org/arachismine/begin.do). Expression (FPKM) values were examined across different tissues, and transcription factors showing expression (FPKM > 0) in reproductive tissues (seed and pod) were selected for heatmap visualization using the online tool ClustVis (https://biit.cs.ut.ee/clustvis/). To identify regulatory elements in the *Ara h* promoters, 1 kb upstream regions of all four known *Ara h* genes were extracted using genomic sequences available in the Ensembl Plants database. These 1 kb upstream sequences were used as input in the PlantCARE (Cis-Acting Regulatory Elements) database (https://bioinformatics.psb.ugent.be/webtools/plantcare/html/) to identify regulatory motifs in these sequences. Motifs corresponding to candidate genes (CGs) underlying *trans*-PQLs were then identified.

## Results

### Genetic variability in the peanut accessions for major allergen proteins using SDS-PAGE and ELISA

**Fig. 1:**
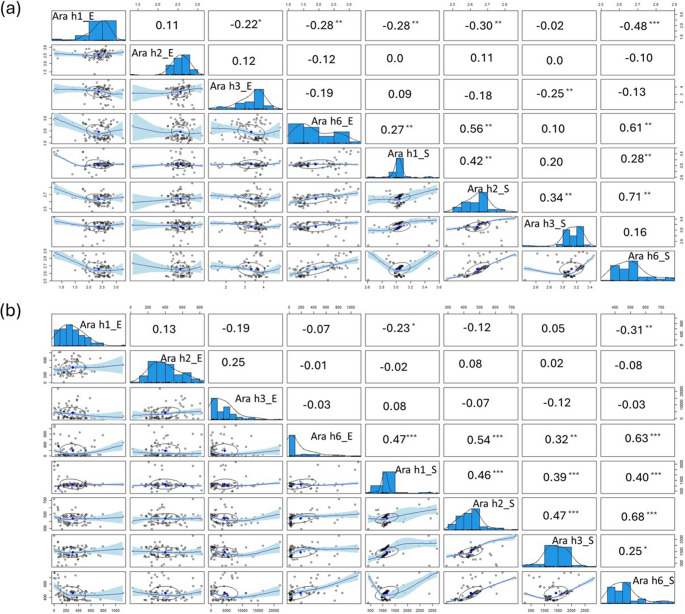
Pearson rank correlations, frequency distribution, and regression curves for (**a**) raw and (**b**) log-transformed Ara h1, Ara h2, Ara h3 and Ara h6 protein data, as estimated using densitometric analysis of SDS-polyacrylamide gels and ELISA. Ara h1_E: Ara h1 estiamted using ELISA; Ara h2_E: Ara h2 estimated using ELISA; Ara h3: Ara h3 estimated using ELISA; Ara h6: Ara h3 estimated using ELISA; Ara h1_S: Ara h1 esitamted using SDS; Ara h2_S: Ara h2 estimated using SDS; Ara h3_S: Ara h3 estimated using SDS; Ara h6_S: Ara h6 estimated using SDS

The data for peanut allergens (Ara h1, Ara h2, Ara h3, and Ara h6) showed substantial variation, as revealed by descriptive statistics, frequency distribution plots, phylogenetic analysis, and Pearson rank correlations (Fig. 1; Tables S1-S4). Descriptive statistics revealed significant differences when calculated using log-transformed protein trait values. This variation was more conspicuous for the densitometric values than for the ELISA estimations. For instance, the CV% for densitometric values ranged from 8% (for Ara h3) to 31% (for Ara h6), whereas it ranged from 2.5% (for Ara h2) to 5.6% (for Ara h3) for ELISA values. Furthermore, when the values for individual proteins were compared, the CV% for densitometric values was highest for Ara h6 (31.14%), followed by Ara h3 (21.48%), Ara h1 (18.73%), and Ara h2 (8.87%). In contrast, for ELISA, the highest value was observed for Ara h3 (5.62%), followed by Ara h1 (4.91%), Ara h6 (3.25%), and Ara h2 (2.59%) (Table S5). The distribution of most protein traits was primarily normal, however, a few traits—such as Ara h3 (estimated using ELISA and densitometry) and Ara h6 (estimated using ELISA)—exhibited skewed distributions. Because of the skewness, all trait data were also subjected to log-transformation, and both the raw and log-transformed datasets were used for the GWAS analysis.

Estimates of Pearson correlations between protein concentrations measured by densitometry (Fig. 1a) and ELISA (Fig. 1b) for all 92 accessions revealed 10 out of 28 possible combinations with significant and positive correlations (Fig. 1a and b). These correlations were more pronounced among different protein types measured using the same method, and less prominent between protein estimates obtained using the two different methods, except for a positive correlation observed between Ara h6 estimates based on densitometry and ELISA.

However, when correlations were calculated for the 23 lines selected for UPLC analysis, significant and positive correlations were more evident for the same protein measured using all three methods (densitometry, ELISA, and UPLC; Fig. S2). Furthermore, when correlations across methods were examined, strong correlations among proteins were observed for UPLC-based estimations, ranging from 0.43^**^ for Ara h6_UPLC and Ara h3_UPLC to 0.87^**^ for Ara h6_UPLC and Ara h1_UPLC (^**^ = *P* < 0.01), compared with proteins estimated using ELISA and densitometry. However, even though these correlations are significant, they do not necessarily reflect a causal relationship. For ELISA, highest positive correlation observed was 0.47^**^ between Ara h1_ELISA and Ara h3_ELISA; similarly, for SDS-densitometry, highest correlation was 0.75^**,^ observed between Ara h3_SDS and Ara h6_SDS.

**Fig. 2:**
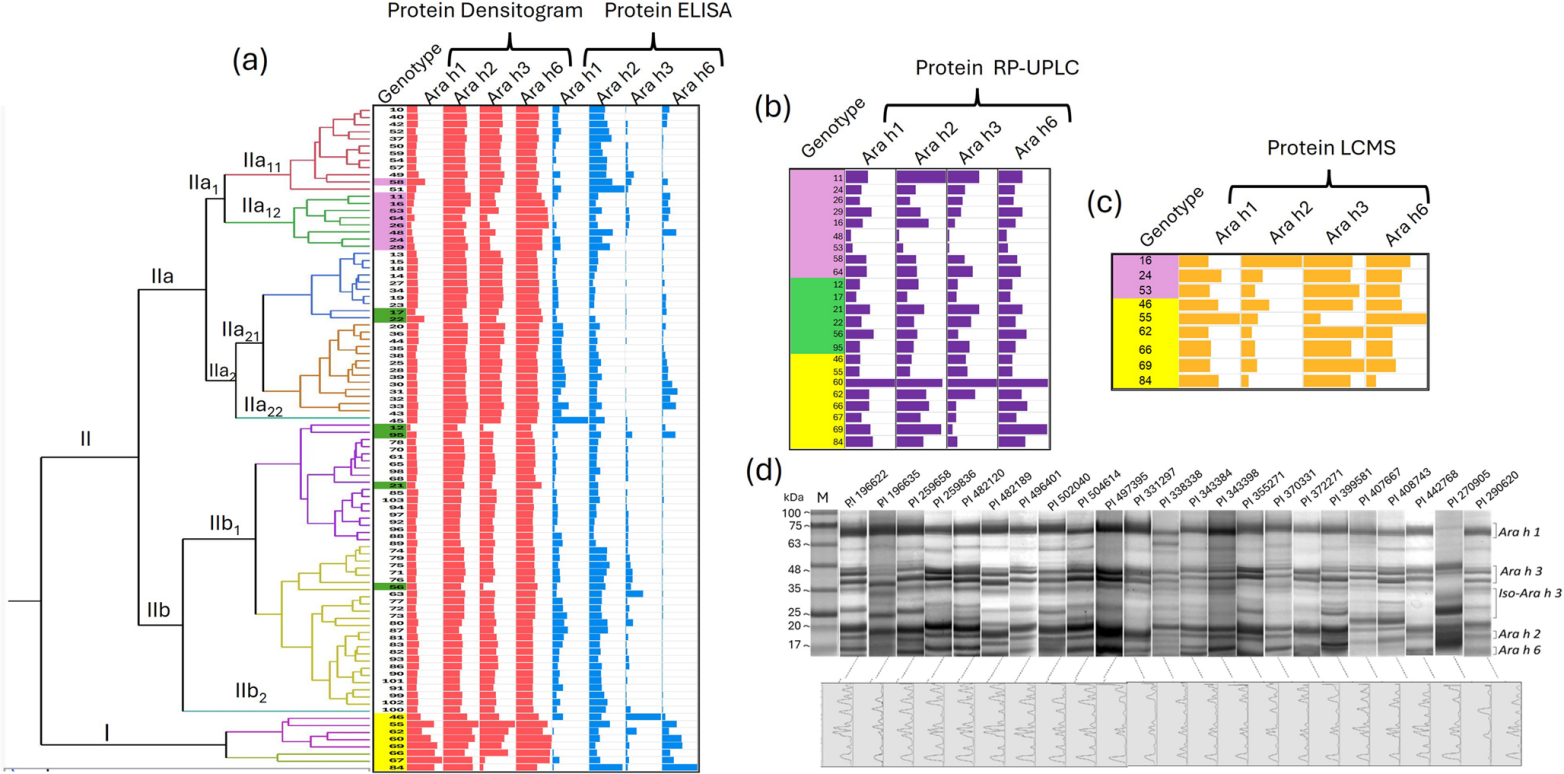
Clustering of peanut genotypes from the U.S. peanut mini-core collection based on estimations of four major allergenic proteins in peanut seeds. (**a**) Phylogenetic analysis and bar diagrams depicting protein concentrations estimated by densitometry and ELISA for the four Ara h proteins across all 92 U.S. mini-core peanut accessions. (**b**) Bar diagrams showing protein abundances estimated using RP-UPLC for 23 selected lines. (**c**) Bar diagrams showing protein quantities estimated using LC-MS for 9 selected accessions. The genotype indicated in pink and yellow are the lines with extreme protein values largely showing stable quantities estimated using different methods. Genotypes with green color are intermediate. These selected lines were also used for UPLC and LC-MS (only 9 lines) analyses. (**d**) Composite image prepared by slicing and assembling gel lanes showing seed storage proteins for 23 selected peanut accessions resolved on 12% SDS–polyacrylamide gels. For original images from which the lanes were cropped, see Figure S4

Overall, positive and significant correlations were more prominent than negative correlations. For the same protein estimated using different methods, correlations were largely positive, except for Ara h2_UPLC vs. Ara h2_ELISA and Ara h2_SDS vs. Ara h2_ELISA, where significant negative correlations were observed (for a possible explanation, see Discussion). Moreover, all four protein estimation methods—densitometry, UPLC, ELISA, and LC–MS—identified the same three genotypes (PI 343384, PI 497395, and PI 259658) as having stable allergenic protein values when compared across methods. A closer examination revealed relatively low and stable values for genotype PI 259658 for proteins Ara h3 and Ara h6 in at least three of the four methods, whereas the remaining two lines consistently showed high values in at least three methods (for protein values, see Fig. 2 and Tables S6).

Phylogenetic analysis and Pearson rank correlations revealed genetic variability among the peanut accessions for the four protein traits. The phylogenetic analysis showed two major clusters: the first cluster consisted of seven peanut accessions, whereas the second cluster was further separated into two sub-clusters (Fig. 2). Overall, the clusters grouped the accessions based on the content of immunogenic proteins; accessions with high and low levels of immunogenic proteins (determined using both methods) were grouped in Cluster I and Cluster IIa 1.2, respectively. This clustering also enabled us to select a subset of 23 accessions for estimating immunogenic protein content using UPLC, and a sub-subset of 9 accessions for estimation using LC-MS. Among the 23 accessions, nine showed low protein concentrations using both methods (UPLC and LC-MS), eight accessions showed high protein content, and six accessions showed intermediate protein content. Overall, the 23 accessions exhibited largely positive correlations between concentrations of different allergenic proteins determined using different methods, as well as correspondence in values obtained for the same proteins across methods. Similarly, the nine accessions selected for LC-MS showed general correspondence in protein concentrations of allergenic proteins determined using four estimation methods (densitometry, ELISA, UPLC, and LC-MS). The average values for different proteins varied substantially when assessed using different methods, reflecting fundamental differences in these estimation approaches, as each evaluates proteins based on different properties. For instance, denaturing PAGE gels resolve proteins based on molecular weight (linear amino acid sequence or 1D structure), ELISA determinations rely on antibody binding to epitopes (3D protein structure), UPLC resolves proteins based on hydrophobicity (influenced by 1D, 2D, and 3D structure), and LC-MS further resolves proteins based on mass, with quantitative estimates made using relative or absolute methods. Between the two methods, densitometry and ELISA, the average values of the four Ara h proteins ranged from 443.18 µg/g to 1421.07 µg/g using densitometry and from 198.75 µg/g to 5316.81 µg/g using ELISA, indicating substantially higher variability in the ELISA measurements compared to densitometry.

### Marker trait associations

**Fig. 3:**
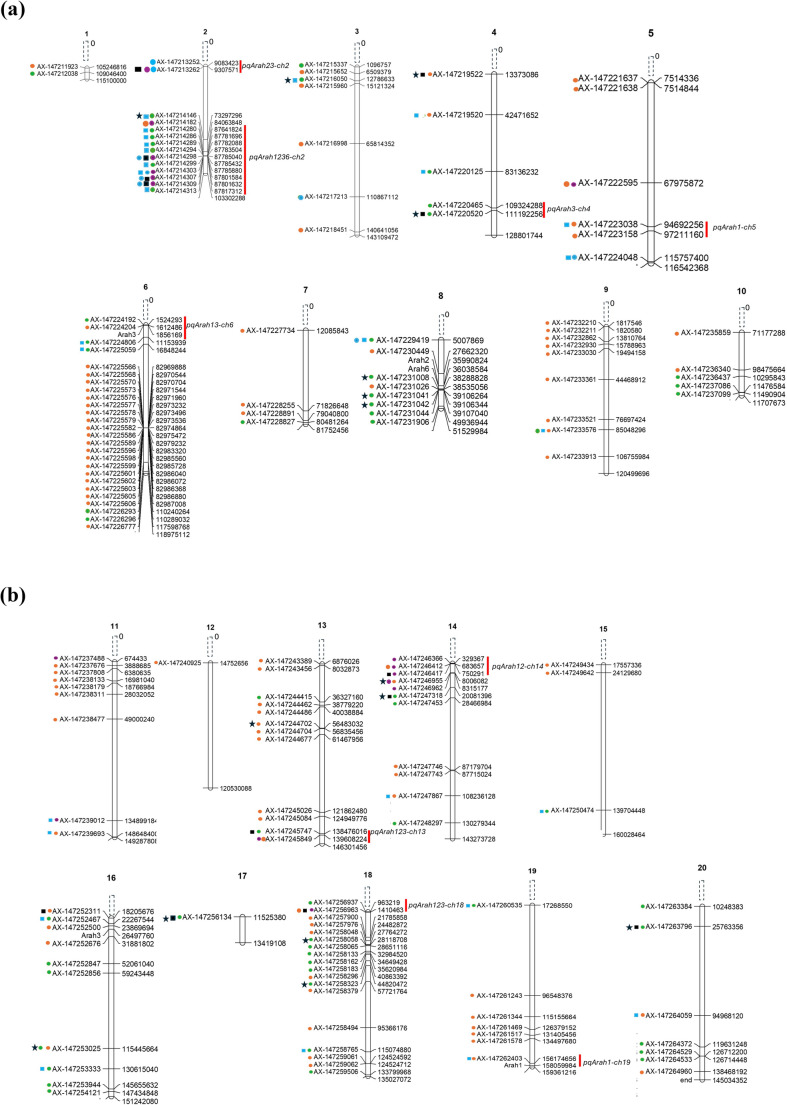
A physical chromosome map of all the MTAs identified using both the methods, (FarmCPU and BLINK) with raw and log-transformed data., (**a**) chromosome map for the first 10 chromosomes, (b) chromosome map for chromosomes 11 to 20. Ara h1; Ara h2; Ara h3; Ara h6 MTA identified using log-transformed data; Common MTA using raw data and log-transformed data MTA passing Bonferroni threshold

**Fig. 4:**
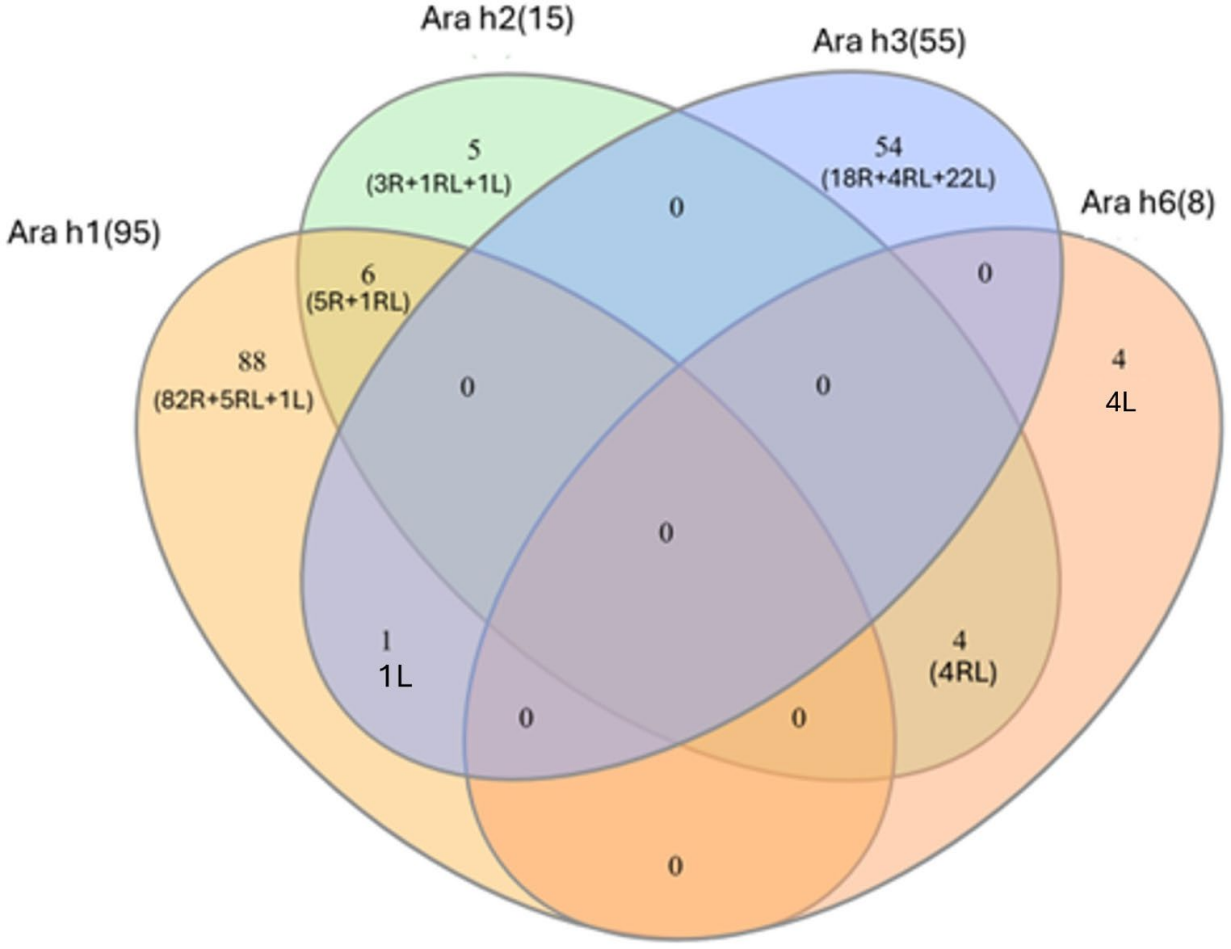
A Venn diagram showing the distribution of MTAs identified for all four protein estimations (using both ELISA and densitogram). The text written in parenthesis indicate the individual MTAs identified using GWAS for raw (R), log-transformed (L) and raw+log transformed (RL) protein values.

A total of 165 MTAs were identified for the four Ara h proteins using the BLINK and FarmCPU methods. Of these 165 MTAs, 124 were uniquely identified using raw data, 28 were identified using log-transformed data, and the remaining 13 were identified using both data types (Figs. 3a and b; Fig. 4; Table 1). In total, 90 MTAs (raw + log-transformed data) were identified using BLINK, while the remaining 118 MTAs were identified using FarmCPU; 43 MTAs were common between the two methods. When MTAs identified using BLINK were compared across the two data types, 76 were detected using raw data and 24 using log-transformed data, with 10 MTAs common between the two. Similarly, using FarmCPU, 90 MTAs were identified from raw data and 41 from log-transformed data, with 13 MTAs common to both data types. Thus, in total, there were 13 MTAs (both using FarmCPU and Blink) which were commonly identified using raw data and log-transformed data (Fig. 1a, b; Table 1). These MTAs belonged to different traits as follows: 4 MTAs for both *Ara h2* and *Ara h6* on chromosome 2, 4 MTAs for *Ara h3*, one each, on chromosomes 4, 13, 17 and 20, 2 for *Ara h1* on chromosomes 4 and 16, 1 for Ara h2 on chromosomes 14 and 1 MTA was commonly identified for *Ara h1* and *Ara h2* on chromosome 18, 16 MTAs were commonly identified using both raw data and log-transformed data (Fig. 3a, b; Table 1).

**Table 1:** List of MTAs that either met the Bonferroni threshold or were commonly identified through GWAS using both raw and log-transformed data. Ch: Chromosome

Type of data	Trait	Marker ID	Allele (Ch)	Position
Raw data (bonferroni qualified)	Ara h1(densitometry)	AX-147,219,522	A/G(4)	13,373,086
		AX-147,231,042	C/T(8)	39,106,343
		AX-147,245,747	T/G(13)	138,476,009
		AX-147,253,025	C/A(16)	115,445,668
	Ara h3(ELISA)	AX-147,220,520	T/C(4)	111,192,257
		AX-147,231,008	T/C(8)	38,288,829
		AX-147,231,041	T/C(8)	39,106,266
		AX-147,244,702	T/C(13)	56,483,033
		AX-147,246,955	C/T(14)	8,006,082
		AX-147,258,058	G/T(18)	28,118,708
		AX-147,258,323	A/C(18)	44,820,474
Log transformed (bonferroni qualified)	Ara h3 (ELISA)	AX-147,216,050	G/A(3)	12,786,633
		AX-147,247,318	T/G(14)	20,081,395
		AX-147,214,146	G/A(2)	73,297,297
		AX-147,256,134	T/G(17)	115,253,809
	Ara h3 (densitometry)	AX-147,263,796	G/A(20)	25,763,355
	Ara h1(ELISA)	AX-147,223,038	T/C(5)	94,692,256
Common in raw data and log-transformed data (*P* < 0.001)	Ara h1(SDS)	AX-147,252,311	G/A (16)	18,205,677
		AX-147,245,747	T/G(13)	138,476,009
		AX-147,219,522	A/G(4)	13,373,086
	Ara h2(ELISA)	AX-147,256,963	T/C(18)	1,410,463
	Ara h2/Ara h6	AX-147,214,309	C/T(2)	87,801,628
		AX-147,214,307	A/C(2)	87,801,580
		AX-147,213,262	A/G(2)	9,307,571
		AX-147,214,298	T/C(2)	87,785,041
	Ara h2(densitometry)	AX-147,246,417	A/G(14)	750,291
	Ara h3 (densitometry)	AX-147,263,796	G/A(20)	25,763,355
	Ara h3(ELISA)	AX-147,247,318	T/G(14)	20,081,395
		AX-147,220,520	T/C(4)	111,192,257
		AX-147,256,134	T/G(17)	115,253,809

### Distribution of MTAs for different proteins

For the four major allergenic proteins, the distribution of MTAs was as follows: (i) 95 MTAs for Ara h1, 15 MTAs for Ara h2, 55 MTAs for Ara h3, and 8 MTAs for Ara h6. (ii) Six MTAs were commonly identified between Ara h1 and Ara h2, of which five were identified using raw protein data and one was identified using both raw and log-transformed data. One common MTA was identified between Ara h1 and Ara h3 using raw data, and four common MTAs were identified between Ara h2 and Ara h6 using both raw and log-transformed data (Fig. 4). The QQ plots for MTAs identified using BLINK and FarmCPU are presented in Figure S3. MTAs for Ara h proteins were distributed on all the 20 chromosomes with maximum number of MTAs on chromosome 6 (22) and minimum of 1 each on chromosomes 13 and 17 (Fig. 3a, b).

### MTAs mapped in ***cis*** and ***trans*** to the structural ***Ara h*** genes

Assignment of the MTAs identified in the present study to the peanut genome, relative to the known locations of the *Ara h1*, *Ara h2*, *Ara h3*, and *Ara h6* genes (including their homoeologous copies), revealed that five of the eight genes map in proximity to MTAs. These genes include *Ara h3* on chromosomes 6 and 16, *Ara h2* and *Ara h6* on chromosome 8, and *Ara h1* on chromosome 19. These genomic regions were examined in more detail by considering LD on these chromosomes to assess whether the MTAs fall within the LD blocks surrounding these genes. Such MTAs were classified as *cis*-PQLs (QTLs associated with known genes). Following this criterion, *cis*-PQLs were identified for two of the five *Ara h* genes (*Ara h3* on chromosome 6 and *Ara h1* on chromosome 19), while the remaining three genes, i.e. *Ara h2*, *Ara h6*, and *Ara h3* (chromosome 16)-were mapped in the proximity to MTAs but did not fall within the LD blocks. *Ara h3* (chromosome 16) was located 268.8 kb from the LD block. Interestingly, *Ara h2* and *Ara h6*, both located on chromosome 8, were found to be in LD with each other and therefore may be co-regulated by the same PQL. Details on LD decay distance and the *cis*- and *trans*-PQLs are provided in Table S7.

Additionally, six *trans*-PQLs (excluding loner MTAs that do not fall close to other MTAs in LD) were identified that may be involved in regulating the expression of *Ara h* genes. These *trans*-PQLs included *pqArah223-ch2*, *pqArah3-ch4*, *pqArah1-Ch5*, *pqArah12-ch14*, and *pqArah123-ch18*. While two PQLs controlled a single *Ara h* gene, the remaining three controlled more than one *Ara h* gene and can therefore be considered pleiotropic PQLs (Table S6). These PQLs were further examined for candidate genes (CGs).

### CGs controlling Ara h proteins and in silico expression analysis

The PQLs were further investigated to identify the underlying candidate genes. Although several protein-coding genes were found to within the PQL regions, we focused on transcription factors and genes encoding proteins similar to the Ara h proteins. Thirteen genes showing expression in developing seeds, based on in silico analysis, were identified within the PQL regions; these genes encoded transcription factors or Ara h–like proteins. These 13 CGs were located in PQL regions on chromosomes 4, 5, 13, 14, and 18. The transcription factors identified included FAR1, winged double-helix DNA-binding protein, two different ethylene-responsive TFs (ERFs), a NAC TF, two different bHLH TFs, a bZIP TF, a cupin (Ara h-like protein), a homeobox-lucine zipper protein, a TATA-binding module, and a GATA TF.

**Fig. 5:**
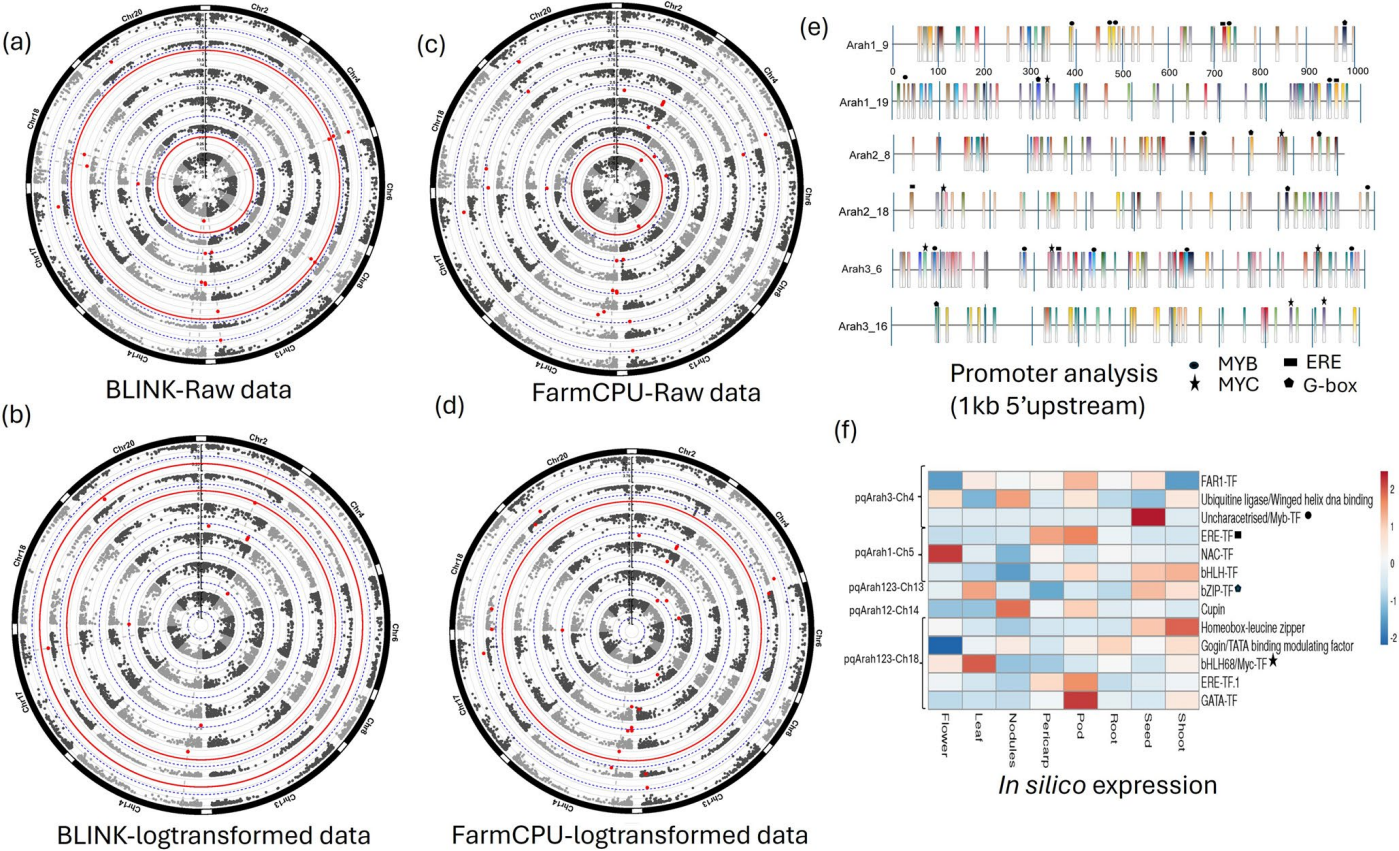
Genomic distribution of PQLs, identification of candidate transcription factors, and promoter analysis of *Ara h* genes for TF binding sites. (**a**–**d**) Circular Manhattan plots showing the positions of important MTAs using (**a**) BLINK with raw values, (**b**) BLINK with log-transformed values, (**c**) FarmCPU with raw protein values, and (**d**) FarmCPU with log-transformed protein values. Only MTAs involved in PQLs and those passing the Bonferroni threshold are shown in these Manhattan plots. The order of tracks in each plot, from innermost to outermost, is as follows: Ara h1 protein estimates using ELISA, Ara h1 estimates using SDS-PAGE, Ara h2 estimates using ELISA, Ara h2 estimates using SDS-PAGE, Ara h3 estimates using ELISA, and Ara h3 estimates using SDS-PAGE. The suggestive threshold (P < 0.001) is indicated by blue broken lines, and the Bonferroni threshold is indicated by red solid lines in each case. MTAs involved in PQLs or those passing the Bonferroni threshold are highlighted with red bullets. (**e**) Promoter analysis of the 5′ upstream regions of the*Ara h1*, *Ara h2*, and *Ara h3* genes showing key transcription factor binding sites (regulatory motifs) associated with TFs identified in the PQLs. Only *Ara h* genes involved in the PQLs are included in this analysis; therefore, motifs for *Ara h6* are not included. (**f**) Heatmap showing the *in silico* expression analysis of transcription factors and Ara h-like proteins (Cupin) underlying the PQLs. The text on the right side indicates the names of TFs, and the text on the left indicates the names of PQLs. Fragments per kilobase per million (FPKM) values extracted from *in silico* datasets were used to generate the heatmap. Expression values for the same tissue from different experiments were averaged prior to heatmap construction

A gene encoding a MYB TF showed high expression exclusively in seeds, whereas the remaining genes were also expressed in other tissues (flower, leaf, nodules, pericarp, pod, root, and shoot) in addition to seeds (Fig. 5). However, five genes—FAR1, both ERF transcription factors (ERF-TFs), a bHLH transcription factor, a TATA-binding module transcription factor, and a GATA transcription factor—showed relatively high expression in reproductive organs (pod and/or seed) compared with vegetative tissues.

### Regulatory motifs in the 1 kb upstream regions of the ***Ara h*** genes

Out of the 13 CGs identified in the PQLs for Ara h1, Ara h2, and Ara h3, binding sites for MYB, MYC, ERF, and bZIP TFs were identified in the 5′ upstream regions of all three *Ara h* protein-coding genes. For the bZIP TF, the corresponding motif known as the G-box was identified (Fig. 5). The maximum (9; five for MYB, two for MYC, and one for ERF) and minimum (3; one for G-box and two for MYC) numbers of binding sites were identified in the upstream regions of *Ara h3* on chromosomes 6 and 16, respectively. Among the remaining genes, the upstream regions of *Ara h1* (on chromosomes 9 and 19) and *Ara h2* on chromosome 8 each contained five binding sites, whereas *Ara h2* on chromosome 18 contained four binding sites. Overall, MYB binding sites were the most abundant (12), followed by MYC (8), and G-box and ERF motifs (five each) across all *Ara h* promoter regions. 

## Discussion

Breeding peanuts for reduced allergenicity is challenging due to the involvement of multiple targets, the biochemical nature of the phenotype, its expression at a terminal stage of plant development, and the lack of DNA markers to guide the selection of genotypes with the desired protein phenotype. Although the major peanut allergen genes were cloned long ago (*Ara h1*: Viquez et al. 2003; *Ara h2*: Viquez et al. 2001; *Ara h3*: Rabjohn et al. 1999; *Ara h6*: Suhr et al. 2004), the genetic regulation of allergen accumulation in peanut remains largely unknown. While *Ara h2* and *Ara h6* both encode 2 S albumin trypsin inhibitors, the other two major allergens—Ara h1 and Ara h3—show homology to 11 S seed storage proteins and plant vicilins (members of the cupin family), respectively.

Peanut allergen genes belong to a complex multigene family, and the major allergens (Ara h1, Ara h2, Ara h3, and Ara h6) are distributed across six different chromosomes. Several isoforms of these genes are also known in peanut (Ratnaparkhe et al. 2014; Bernard et al. 2007). As stated earlier, the genetic regulation of the accumulation of major allergenic proteins in peanut is underexplored. However, similar studies identifying PQLs for allergen and quality proteins have been reported in wheat (*Triticum aestivum*) and pea (*Pisum sativum*) (Bourgeois et al. 2011; Zhou et al. 2021; El Hassouni et al. 2023). In these studies, wheat proteins were estimated using RP-HPLC (Zhou et al. 2021) and LC–MS (El Hassouni et al. 2023), whereas pea proteins were analyzed using fast protein liquid chromatography (FPLC) and 2D electrophoresis (Bourgeois et al. 2011).

In the present study, we attempted for the first time to identify the PQLs controlling the production and accumulation of major peanut allergen proteins (Ara h1, Ara h2, Ara h3, and Ara h6). Initially, the U.S.-peanut mini-core accessions were screened using SDS-PAGE and ELISA, followed by RP-UPLC and LC-MS analyses of selected accessions. Some information on genetic regulation of these proteins is available from an earlier study involving temporal and spatial gene expression analysis in seeds (Kang et al. 2007a, b). In that study, the expression patterns of *Ara h3* were found to be more variable compared to the other major allergenic proteins. Furthermore, Ara h1 and Ara h3 mRNA levels were more abundant in cotyledons compared to embryonic axis. In another study, gene families encoding major peanut allergenic proteins were examined in detail by Ratnaparkhe et al. (2014). This study revealed that genes for major peanut allergenic proteins are interspersed with many low-copy genes and transposable elements, and the epitopes for these proteins are less conserved between different plant species.

We used densitometric analysis of SDS polyacrylamide gels and ELISA measurements of the major allergenic proteins as major phenotypic traits for PQL identification. These methods are widely used for protein quantification in various crop plants, including peanut (Rustgi et al. 2022; Koppelman et al. 2016; Wu et al. 2016; Cong et al. 2008) and cereals such as wheat (Rustgi et al. 2022; Wang et al. 2021). Although it is useful to make estimations using multiple methods that resolve, identify, and quantify proteins based on different physicochemical properties, these methods show differences in absolute values. Nonetheless, general trends in protein estimates remain consistent across methods. ELISA relies on specific antigen-antibody interactions and thus estimates protein abundance based on the 3D structure, which determine the accessibility of epitopes (Hands et al. 2020). In contrast, densitometry measures the total protein mass represented in a particular band resolved by SDS-PAGE, which is based on protein molecular weight or 1D structure (Zimmer et al. 2021). Therefore, technical differences between these methods, such as antibody-antigen interaction, protein composition, and protein size, can result in observed differences in the determined content of immunogenic proteins (Rustgi et al. 2019; Mejías et al. 2014). Given this knowledge, it is possible to interpret the GWAS results, in which only four markers were found to be common between proteins estimated using ELISA and densitometric analysis. However, most of the unique MTAs from both methods were located in the same PQL regions based on LD, indicating that these MTAs are identifying the same genomic region (Fig. 4).

By utilizing SDS-PAGE as a preliminary screening method, followed by ELISA and UPLC to analyze the U.S. peanut mini-core collection, we observed significant diversity in the contents of Ara h1, Ara h2, Ara h3, and Ara h6, as evidenced by the wide protein concentration range for each protein in this collection. The protein data obtained from these methods were subsequently used for phylogenetic analysis. Similar variability in other seed compositional traits, such as oil, fatty acid, flavonoid, and resveratrol content, has also been reported (Wang et al. 2013). The diversity in allergen content, as revealed by ELISA and UPLC (Fig. 2b, c), indicates that the genetic variation necessary to breed for reduced allergen content exists within the U.S. peanut mini-core collection. Therefore, this germplasm collection appears suitable for mapping the regulators of observed differences in allergen content and for developing molecular markers for this complex trait. The present study represents one such effort, in which potential TFs underlying the *trans*-PQLs identified through our GWAS analysis have been identified.

Two different data types for each protein quantification method were used for identifying MTAs: the actual raw data and the log-transformed values of protein estimates obtained through ELISA and densitometry. Some lines (e.g., PI 497318 and PI 343384 for Ara h1, PI 337399 and PI 259836 for Ara h2, PI 497395 and PI 493631 for Ara h3, and PI 270905 and PI 276235 for Ara h6) in our GWAS panel showed significantly different protein values from the remaining lines and such entries are generally excluded from GWAS analysis. However, removing such data points may adversely affect the results because the highly diverse genotypes in the U.S. peanut mini-core may carry unique alleles that contribute positively or negatively to the trait. Therefore, we did not remove these values from the analysis. Recognizing the possibility that these extreme values could confound GWAS results and lead to false positive MTAs, we also used log-transformed values for the protein traits, which improved the trait distributions, as shown in Fig. 1b. Similarly, two different criteria were used for identifying significant MTAs: (i) Bonferroni threshold and (ii) *P* < 0.001. We acknowledge that suggestive *P* < 0.001 is less stringent and may lead to false positives, whereas highly stringent Bonferroni correction may be over conservative and result in missing important true associations (Rahnenfuhrer et al. 2023). Therefore, we used both criteria and exercised caution when selecting important MTAs. Only the MTAs that were commonly identified using both raw and log-transformed data were considered important. These MTAs were subsequently used to identify the PQLs (based on LD information). In total, we identified six *trans-*PQL and four *cis-*PQLs. The detection of *cis-*PQLs further validates our approach.

Chromosome distribution of MTAs reveals interesting patterns: (i) chromosome 6 carries 22 MTAs for Ara h1 and also contains the *Ara h3* gene; (ii) chromosome 18 carries nine MTAs for Ara h3; and (iii) chromosome 2 appears to carry MTAs for both Ara h2 and Ara h6. This clearly indicates that these proteins are co-regulated in trans by common TFs and may share regulatory elements in their promoter regions. Transcription factors are the most likely candidates; however, other mechanisms—such as epigenetic regulation (including non-coding RNA genes, DNA methylases and demethylases, chromatin-remodeling enzymes, and protein-assortment and degradome machinery) or novel regulatory pathways—may also be involved and require further investigation. For instance, a recent study in soybean, another important crop that causes allergic reactions in humans and production/companion animals, reported that high expression of an abscisic acid biosynthesis gene encoding the 9-cis-epoxycarotenoid dioxygenase 5 enzyme was responsible for the high accumulation of seed storage proteins (Liu et al. 2025). This suggests that more than one mechanism may be involved even in peanut seed storage protein synthesis and accumulation. However, in this study, we focused only on TFs and identified several candidates showing high in silico expression in seeds.

Overall, when the MTAs for each of the four proteins were compared, many more MTAs were identified for Ara h1 (95) and Ara h3 (55) than for the other two major allergens (15 for Ara h2 and 8 for Ara h6), indicating the complex genetic regulation of these proteins. Four common MTAs were identified for Ara h6 and Ara h2 (AX-147214309, AX-147214307, AX-14713262, and AX-147214298), all of which were located on chromosome 2, and six common MTAs were identified for Ara h2 and Ara h3. However, between Ara h1 and Ara h3, only a single common MTA (AX-147253025) was identified. Ara h proteins are known to be highly complex, with several isoforms described for different proteins, a pattern confirmed both by a recent study (Marsh et al. 2022) and by the SDS-PAGE analysis reported here. Marsh et al. (2022) documented two isoforms each for Ara h1, Ara h2, and Ara h6, and at least 11 isoforms for Ara h3, although some of these isoforms were presumed to be truncated proteins produced by five different *Ara h3* genes. Furthermore, at the sequence level, Ara h2 and Ara h6 share 59% identity and have somewhat similar allergenic properties. Therefore, the common MTAs identified for these proteins could partly be explained by similar mode of regulation for these closely related genes. Interestingly, three of the four common MTAs also belong to the same PQL (*pqArah1236.Ch2*). Likewise, Ara h1 and Ara h3 belong to the cupin superfamily and may share regulatory mechanisms, which is consistent with the identification of *cis*-PQLs for Ara h1 in proximity to the *Ara h3* gene on chromosome 6.

GWAS results identified at least two *trans*-PQLs for Ara h3 on chromosomes 4 and 20, which carried genes encoding winged helix DNA-binding proteins, Myb, nucleic acid-binding OB-fold and FAR DNA-binding TFs, as well as co-chaperones such as Dnaj (Hsp40), revealing the complex trans-regulation of major allergenic peanut proteins. Overall, it appears that genetic regulation of the *Ara h* genes is much more complex than previously anticipated, and a detailed analysis is still needed to fully understand their regulation. To date, no study in peanuts has comprehensively examined the regulation of Ara h protein expression, although protein profiling has been conducted in detail for all Ara h proteins. In wheat, the regulation of gluten proteins—which are responsible for gluten sensitivity and allergies—is similarly complex (Merlino et al. 2023; Plessis et al. 2023; Wen et al. 2022; Che et al. 2025). For example: (i) The expression of genes encoding grain storage proteins (low- and high-molecular-weight glutenins and gliadins) is regulated by a TF known as *TaSAD* (scutellum- and aleurone-expressed DOF). This TF binds to *cis* motifs in the grain storage protein (GSP) promoter sequences to regulate gene expression (Merlino et al. 2023). (ii) Another study demonstrated that DNA demethylation, mediated by DEMETER and DRE, plays a key role in the transcriptional activation of GSPs (Wen et al. 2022). A detailed discussion on the genetic regulation of wheat gluten proteins can be found in Che et al. (2025), and a comparison of the regulation of GSPs is provided in Yang et al. (2023).

Interestingly, out of the five PQLs, three (*pqArah1236.Ch2*,* pqArah123.Ch2*,* and pqArah23.Ch18*) were found to control more than one Ara h protein, suggesting that these proteins may be regulated by common transcription factor(s). However, further investigation is needed to elucidate the exact mechanisms through which these proteins are co-regulated and controlled by these PQLs. In peanut, genomic regions have been reported that regulate fatty acids, where MTAs for both oleic acid and linoleic acid were identified using GWAS. Similarly, QTLs controlling multiple seed-related traits have also been discovered, although none have been reported specifically for allergen content.

We also examined the promoters (1 kb upstream of the TSS) of the *Ara h* genes corresponding to the five PQLs to identify binding sites for candidate TFs with seed-specific expression patterns underlying these PQLs (Fig. 4). The binding sites establish cross-correspondence between the *Ara h* genes and the TFs or regulatory *trans*-PQLs. Mainly MYB, MYC/bHLH, ERF, and bZIP (binding to G-box) TFs had binding sites in the promoter regions of the *Ara h1*, *Ara h2*, and *Ara h3* genes. Therefore, it can be speculated that the regulation of Ara h proteins is quite complex and occurs through a network mainly involving TFs as major regulatory genes along with other downstream pathway genes. We further hypothesize that once the role of these TFs in regulating Ara h proteins is confirmed through knockdown or overexpression studies, mutants for these transcription factors could help develop peanut lines with reduced Ara h protein levels.

The above TFs have long been known to play roles during seed development in peanut as well as in other dicots such as soybean (Zhang et al. 2013; Du et al. 2012; Wang et al. 2019, 2023). Similarly, the role of ERF in seed germination and early seedling development has been reviewed in detail by Ahammed et al. (2020). In fact, the regulation of allergen proteins by transcription factors and other regulatory genes is not new and has already been well explored in wheat (Che et al. 2025). For instance, in wheat it has been demonstrated that grain seed storage proteins (similar to Ara h proteins in peanut) are regulated by a complex gene network mediated by TFs such as prolamin box binding factors (PBF) and Scutellum and Aleurone expressed DOF (*TaSAD*) (Dong et al. 2007; Diaz et al. 2005). In that study, transient gene expression assays demonstrated that the expression of these two TFs activates the expression of GSPs. Therefore, wheat lines with silenced or mutated TFs could help produce wheat lines with reduced immunogenicity (Moehs et al. 2019). Similar to our hypothesis based on promoter analysis and in silico expression analysis in peanut, it has been reported in wheat that a seed-specific bZIP protein known as SPA is involved in activating the transcription of low molecular weight glutenin subunit genes (LMW-GS) by binding to the GCN4-like motif (GLM) in their promoters (Albani et al. 1997). In another study, heterodimerization of SPA with SPA Heterodimerizing Protein (SHP) was shown to regulate both HMW- and LMW-GS genes by binding to bZIP-specific *cis*-motifs in the promoters of these genes (Boudet et al. 2019). Similarly, the role of the TaMyb transcription factor in activating the expression of HMW-GS genes was demonstrated in another study (Guo et al. 2015). 

The observed variability in allergen concentrations supports the hypothesis that genetic differences among peanut genotypes significantly influence the expression of allergenic proteins, thereby affecting overall allergenic potential. The distinct allergen expression patterns across accessions highlight opportunities to further characterize peanut germplasm with lower allergenicity. The nine accessions identified using all four protein estimation methods (Fig. 2) represent promising targets for breeding reduced-allergen peanut lines through molecular-assisted crossbreeding. These extreme lines may also serve as parents for developing mapping populations to validate the PQLs identified in this study. Furthermore, we acknowledge the limited number of accessions used in the GWAS analysis, which is one of the limitations of this study. However, despite the limited number of accessions, we attempted to ensure that the selected genotypes captured the complete diversity represented by the 812 lines from which these representative accessions were selected (Otyama et al. 2020). Moreover, despite the limited number of accessions, we were still able to identify two known *Ara h* genes (*Ara h3* on chromosome 6 and *Ara h1* on chromosome 19), as well as three additional genes located in close proximity to the MTAs identified in our GWAS analysis, thereby validating our approach. However, the *trans*-PQLs identified in this study require further validation before they can be implemented in breeding programs. Therefore, mapping experiments using contrasting parental lines for allergen proteins are currently underway in our laboratory to validate the genomic regions identified here (Rustgi, Ingole, Jones, unpublished results). We anticipate that some PQLs will be validated and incorporated into breeding programs aimed at developing low-allergen peanut lines. In addition, the putative transcription factors identified in this study could serve as potential targets for CRISPR-based gene editing to validate their roles in the trans-regulation of Ara h proteins.

## Supplementary Information

Below is the link to the electronic supplementary material.

Supplementary Material 1 (XLSX 33.0 KB)

Supplementary Material 2 (DOCX 1.48 MB)

Supplementary Material 3 (JPG 1.17 MB)

## Data Availability

All the data related to study are either available in the main text or as supplementary information.
