# Supplementary material for: Genetic regulation of major immunogenic protein accumulation in peanut seeds
Source: Funct Integr Genomics. 2026 Jun 11;26(1):132. doi: 10.1007/s10142-026-01916-x (PMC13253668; doi:10.1007/s10142-026-01916-x)
Supplement: Supplementary file 2 — Supplementary Material 2 (DOCX 1.48 MB) [file 10142_2026_1916_MOESM2_ESM.docx]

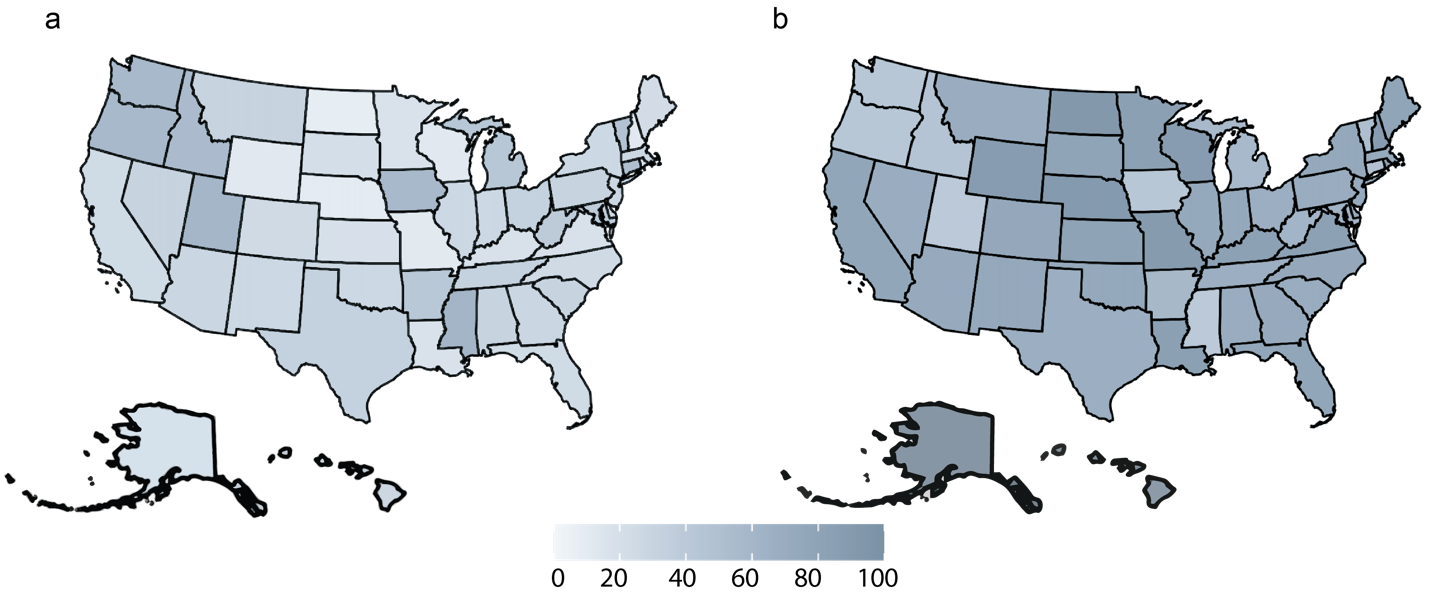


**Figure S1** U.S. maps showing (a) the demographics of individuals with peanut allergy and (b) cases of peanut-induced anaphylaxis. The maps were recreated using data reported by Food Allergy Research and Education (FARE).


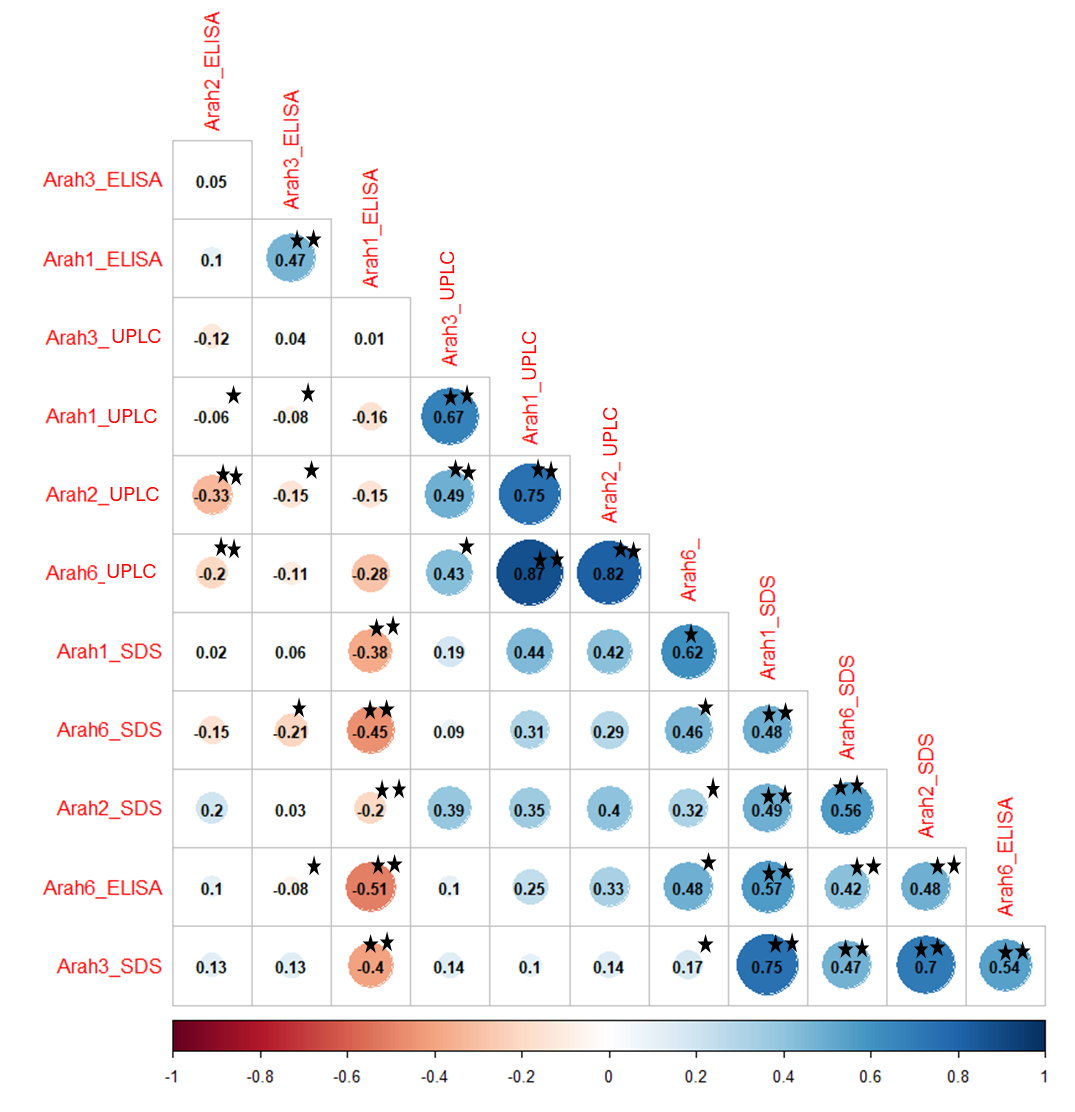


**Figure S2** Correlation plots showing Pearson correlation coefficients among protein estimates for Ara h1, Ara h2, Ara h3, and Ara h6 across 23 selected U.S. peanut mini-core accessions. Arah1_ELISA, Arah2_ELISA, Arah3_ELISA, and Arah6_ELISA represent protein estimates for Ara h1, Ara h2, Ara h3, and Ara h6 obtained using ELISA. Arah1_HPLC, Arah2_HPLC, Arah3_HPLC, and Arah6_HPLC represent protein estimates obtained using HPLC, while Arah1_SDS, Arah2_SDS, Arah3_SDS, and Arah6_SDS represent protein estimates derived from SDS-PAGE–based densitogram analysis. Statistical significance is indicated by superscripts associated with the correlation values: * indicates significance at P < 0.05, and ** indicates significance at P < 0.001. Blue-colored filled circles indicate positive correlations, whereas orange-colored circles indicate negative correlations. The strength of the correlation is represented by the color intensity: darker shades indicate stronger correlations, while lighter shades indicate weaker correlations.


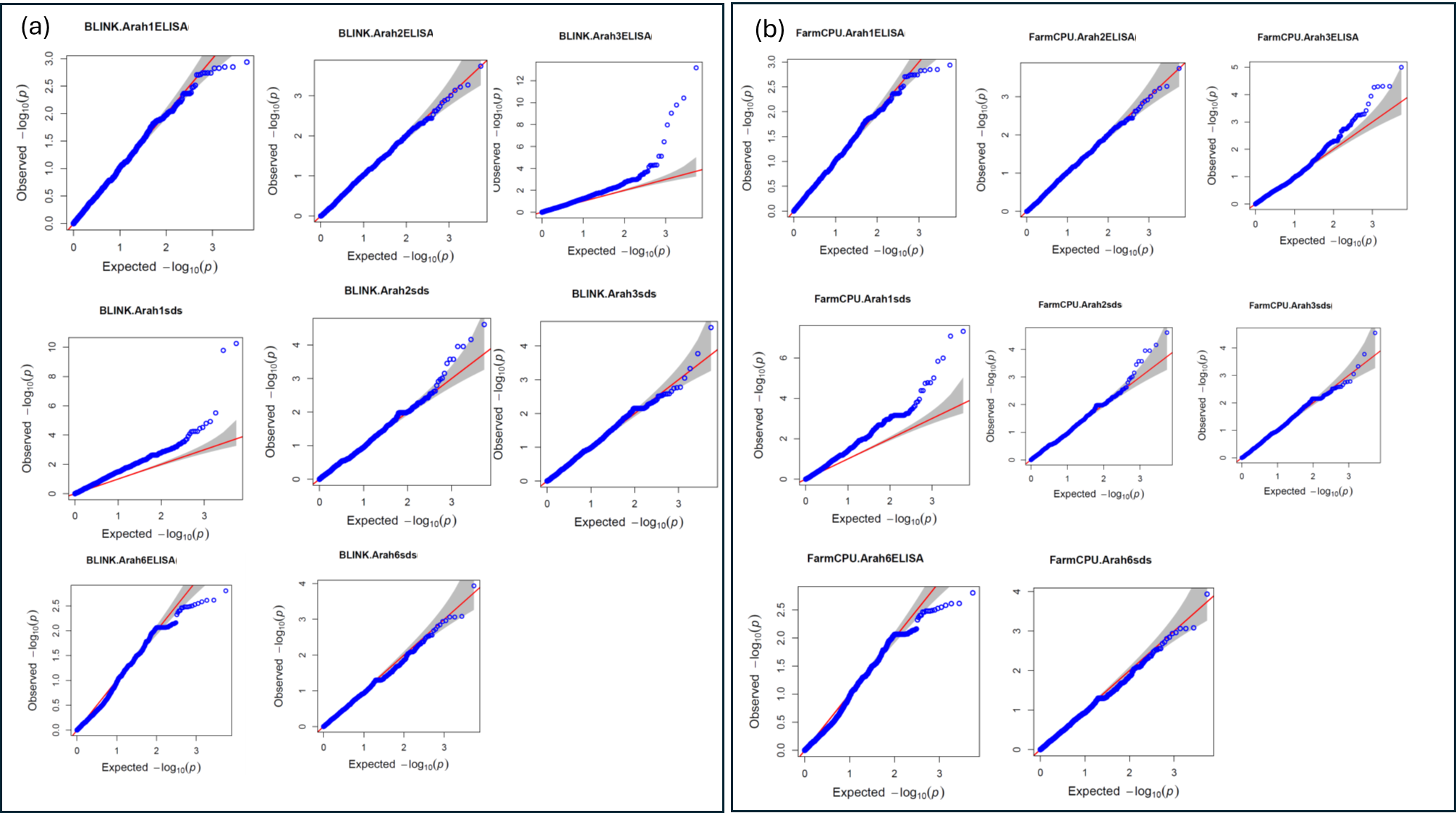


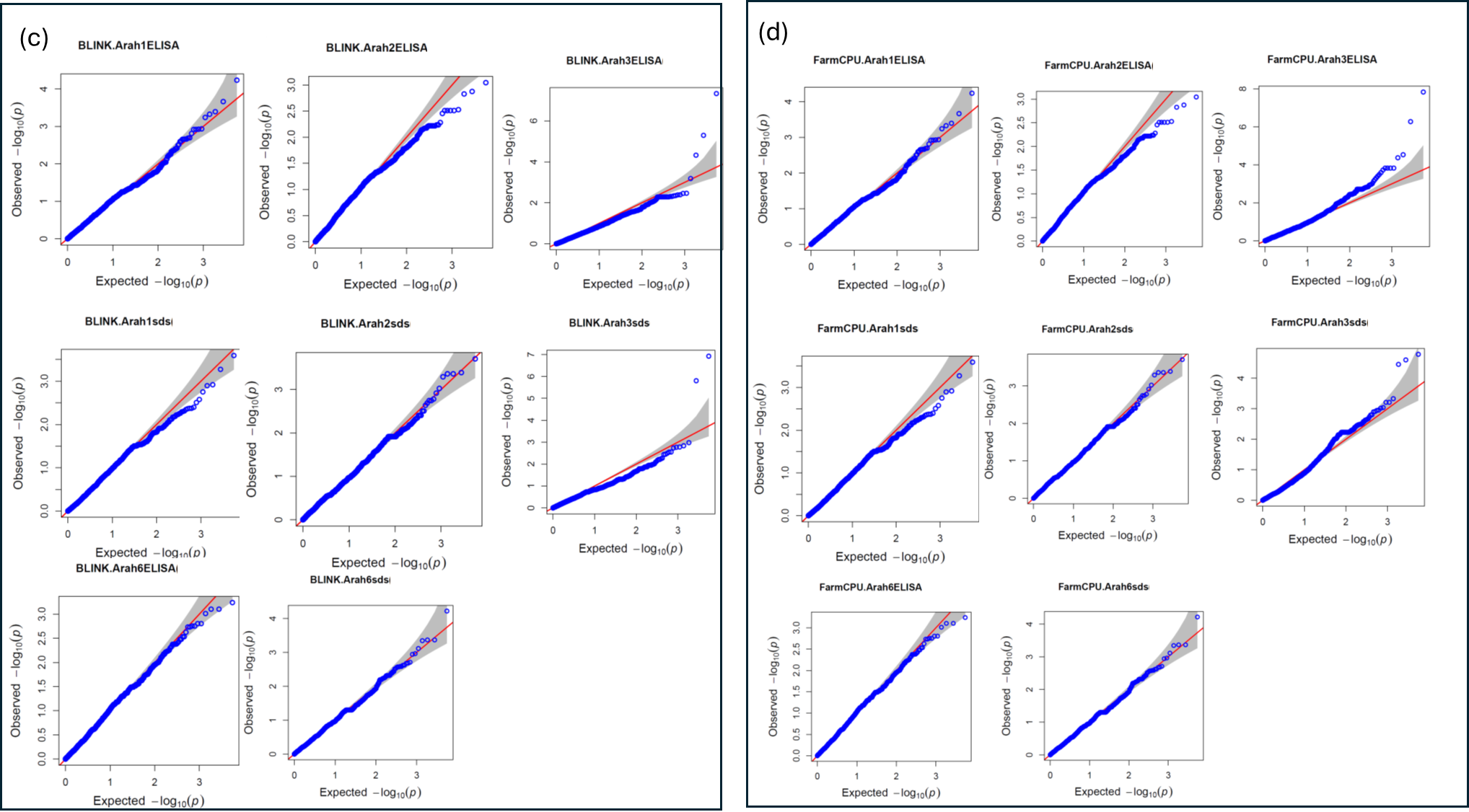


**Figure S3** QQ plots showing the distribution of p-values for marker–trait associations identified using (a) BLINK with raw protein estimates, (b) FarmCPU with raw protein estimates, (c) BLINK with log-transformed protein estimates, and (d) FarmCPU with log-transformed protein estimates.
