# Supplementary figures and images for: Genetic regulation of major immunogenic protein accumulation in peanut seeds
Source: Funct Integr Genomics. 2026 Jun 11;26(1):132. doi: 10.1007/s10142-026-01916-x (PMC13253668; doi:10.1007/s10142-026-01916-x)

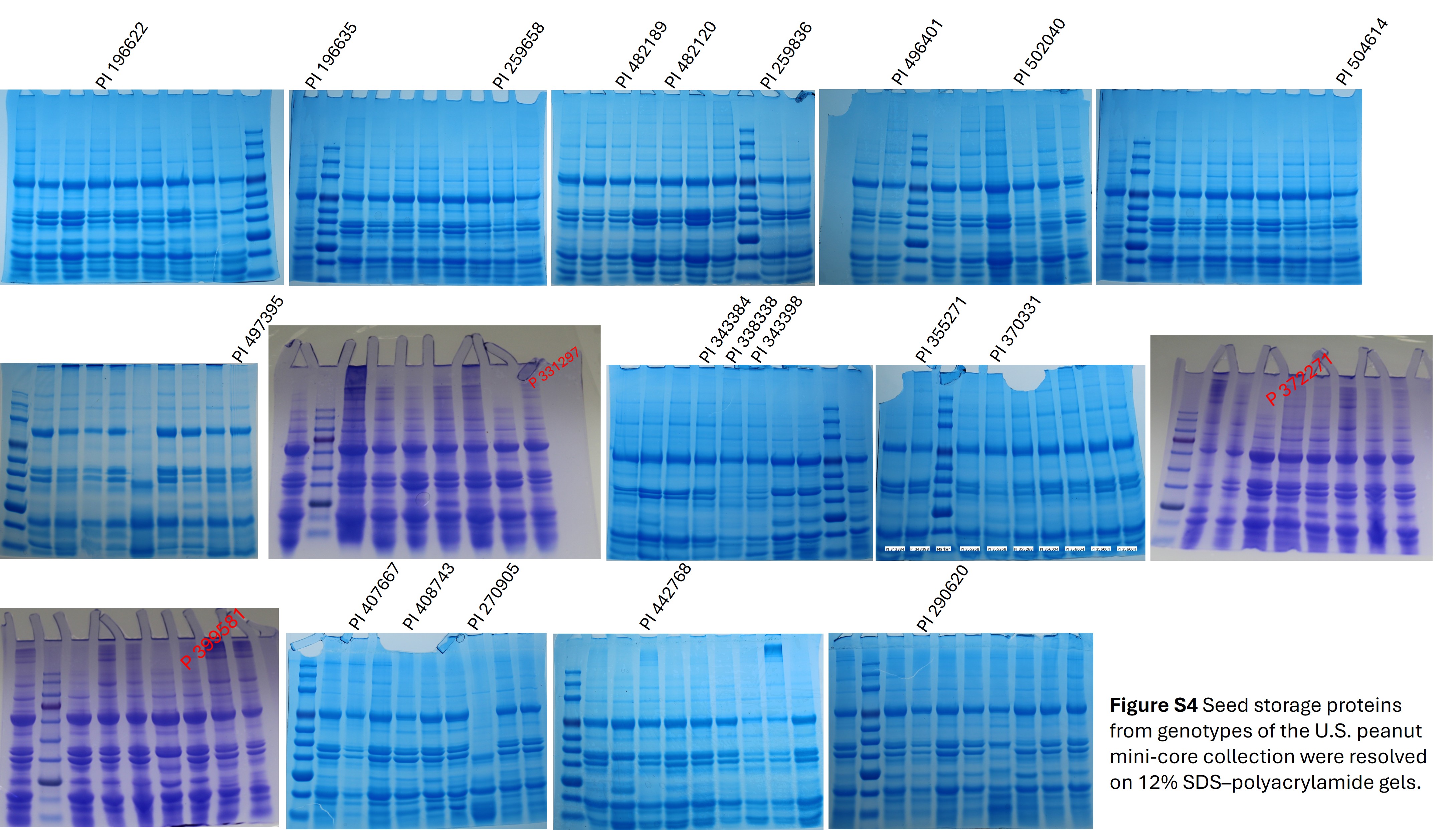

Supplement: Supplementary file 3 — Supplementary Material 3 (JPG 1.17 MB) [file 10142_2026_1916_MOESM3_ESM.jpg]
